# The SPC model as a heuristic tool to better understand autism spectrum disorder and other structural diagnosis in psychiatry and psychotherapy

**DOI:** 10.3389/fpsyt.2026.1805806

**Published:** 2026-09-15

**Authors:** Ludger Tebartz van Elst

**Affiliations:** Department of Psychiatry and Psychotherapy, Medical Center - University of Freiburg, Faculty of Medicine, University of Freiburg, Freiburg, Germany

**Keywords:** autism spectrum disorder, developmental disorders, mental structure, psychotherapy, therapy

## Abstract

Both autism and the broader autism phenotype represent stable mental features over time that often lead to typical psychosocial problem patterns such as bullying, interpersonal conflicts at work and at home, or social withdrawal. These patterns may in turn cause specific temporary medical conditions such as states of tension, agitation, mutism, depression, or psychosis. When presenting to psychiatry or psychotherapy patients generally do not complain about autism, but rather about depression, interpersonal conflicts, and psychosocial difficulties. They may also complain about maladaptive behaviors such as social withdrawal, gaming etc. They often have difficulty understanding themselves, generating a clear sense of self and identity, and understanding other people’s behaviors. The SPC model presented here is a heuristic tool to provide clarity on this complex interplay of different ontological entities. The model analyses life phenomena and developments according to their degree of situational freedom and modifiability. Mental structures are considered to be fateful, fundamental physical phenomena, similar to body height, IQ, and ametropia. Problems and problematic behaviors are not core phenomena of the autistic structure, despite developing in typical psychosocial dynamics and patterns in the biographies of autistic individuals. Examples include bullying, social withdrawal, and dysfunctional communication patterns. Unlike autistic structures, these should not be accepted, but changed. Conditions are understood as specific psychobiological and physical states. Affected persons are not free to intentionally alter such conditions. Unlike bodily structures, they have a beginning and an end, and are transient by nature. Examples include states of tension, depressive episodes and psychotic states. Such conditions should be treated with all available medical and psychotherapeutic means, or endured until they naturally pass. In individual situations, people are not free with respect to their own structure and biological conditions. They cannot manipulate these phenomena voluntarily. They can only compensate or cope. Problems and problem behaviors, however, can and should be solved. These phenomena fall within the realms of freedom and responsibility. Such a comprehensive understanding of one’s own body, mental structure, and psychodynamic mechanisms, as well as the typically evolving interpersonal dynamics, is of utmost importance for the development of a valid and functional self, particularly in autism.

## Introduction

Autism-spectrum-disorder (ASD) belongs to the neurodevelopmental disorders according to DSM-5 (1) and ICD-11 (2) and is characterized by a persistent pattern of deficits in social communication and interaction across different psychosocial contexts (A-criterion in DSM-5, first main criterion in ICD-11) and restricted and repetitive patterns of behaviors, interests or activities (B-criterion in DSM-5, second main criterion in ICD-11). The latter includes sensory peculiarities in terms of hyper- or hyporeactivity to sensory input and unusual interests in sensory aspects of the environment. Like the other neurodevelopmental disorders (intellectual disabilities, ADHD, tic disorders etc.) ASD has long been perceived as a rare condition common only in child and adolescent psychiatry with little relevance for adult psychiatry and psychotherapy (3). This view has changed considerably. Recent prevalence figures are as high as 1-2% (4). The latest figures from the Center for Disease Control estimate the frequency as 1 in 31 children (~3,2%) for 4–8-year-old children assessed in 2022 (5; https://www.cdc.gov/autism/data-research/index.html). These figures had risen again from the 2020 prevalence rate of 1 in 36. Addressing this issue, two recently published, high-profile genetic studies based on extremely large samples demonstrated that increasingly mild manifestations of an autistic phenotype have led to a clinical diagnosis over the past few decades (6). At the same time, two distinct multigenetic clusters were identified: the first was associated with an earlier diagnosis and lower IQ scores; the second was associated with a later diagnosis and increased ADHD and other psychiatric comorbidity (7). These figures illustrate, that ASD in fact is an important issue for adult psychiatry and psychotherapy. Because, since it is a chronic condition, all these children will grow up to be adults with ASD, who often will present to the psychiatric and psychotherapeutic services. Psychiatric comorbidity figures are particularly high for personality disorder diagnoses, affective disorders, anxiety disorders, ADHD, obsessive-compulsive disorders, tic disorders, psychotic disorders and addiction (8–10).

If ASD is a *de novo* diagnosis at time of presentation to psychiatry or psychotherapy this implies that affected patients are rather high functional. I.e. in order to run unrecognized into adulthood affected patients must have had high compensation faculties. And in fact, this section of the ASD population tends to have rather high IQs, average or even above average, and they tend to have acquired high compensation skills. Also, women often run undiagnosed until adulthood for reasons that are not totally clear (11, 12). It might be, the female ASD phenotype differs from the male one, it might be women do better in compensating and camouflaging the core properties of ASD (12–15).

Another issue is that of broader autism phenotype (16). It is well recognized in the scientific literature, that the personality structure of ASD is not a categorical phenomenon but rather presents in a dimensional way. The Autism Quotient (AQ), developed by Baron-Cohen, clearly illustrates this, as the results for the general population follow a normal Gaussian distribution to a large extent (17). This is the reason for coining the term broader autism phenotype which is reserved for people – often parents or relatives of patients – with well recognizable autistic features who however do not fulfil the diagnostic criteria for ASD according to DSM or ICD. Nevertheless, such individuals may experience difficulties due to their ASD traits, whether they are syndromic or sub-syndromic. These traits may be the root cause of interpersonal issues and conflicts in the workplace and relationships, leading to distress and impairment. In such a constellation the sub-syndromal ASD personality property pattern fulfils the qualitative criteria for a personality disorder rather than those of ASD (3). **Box 1** illustrates a typical case in such a constellation.

Box 1 **A case of very high functioning autism with psychiatric relevance.**

At the time of his initial consultation, Mr M., a 43-year-old architect, had been retired for several years. He was referred by his insurance company for an assessment regarding private disability insurance. Even during the initial consultation, his avoidance of eye contact, limited prosody, lack of facial expressions and gestures, and monotonous tone of voice were noticeable. His developmental history reveals that he exhibited slightly delayed motor and language development following an uneventful pregnancy and birth, with no evidence of other early childhood illnesses. He learned to speak late, but then mastered it quickly and perfectly in a peculiar way. His lack of eye contact was noted in kindergarten. At the age of 5, he taught himself to read and developed an interest in words, languages and buildings. He has engaged with these topics excessively throughout his life. He was known as ‘the little professor’ from kindergarten onwards. He has always been diligent, meticulous and very careful, and he probably seemed very formal. However, due to his friendly nature and excellent manners, he was never excluded or bullied. Still, he never had any friends or a romantic relationship. As a child, he often misunderstood proverbs. But he studied them extensively and learned them all. He never played football or any other sports with other children. He was all thumbs and was unable to handle team sports. In any case, he has always been sensitive to noise and avoided crowds, streetcars and busy supermarkets. Having a structured daily routine has always been very important to him. As a child, he would sometimes become very angry when plans changed, but this subsided with time, and he learned to withdraw when he felt stressed. After graduating from high school with good grades, he studied architecture and completed an internship at an architecture firm. The boss was a friend of his father, who is a professor of Oriental Studies. Recognizing his talent, the boss immediately brought him on board at his firm. He had always had a good sense of aesthetics and loved visualizing the inner workings of buildings, as well as planning and optimizing the technical aspects down to the last detail. His boss hired him right after he graduated and quickly made him a partner. Together, they were a perfect team. The boss brought in the contracts, he handled the aesthetic and technical planning, and the boss took care of construction management and financial matters. Together, they became somewhat famous, even winning an architecture award. Other than that, he had always lived solely for his work. He had done well for himself and wanted for nothing. Aside from his family and work, he had no other social contacts. When his boss retired, he was set to take over the company. However, everything changed very quickly and the company went bankrupt within six months. He struggled to get along with clients or construction workers. Communication just wasn’t his strong suit. Everyone did whatever they wanted. Bills went unpaid, construction workers stopped showing up and he couldn’t secure any new contracts. However, the company had already gone bankrupt before the old projects were completed. Under extreme stress, he was unable to speak properly, withdrew into his apartment, stopped opening his mail and completely fell apart. His family doctor sent him to a psychiatrist. The psychiatrist diagnosed him with depression and admitted him to a psychosomatic clinic. It had been a disaster for him there. He couldn’t tolerate the medication; he couldn’t stand all the noise and he didn’t understand what was going on in group therapy. He discharged himself within two weeks and led a totally withdrawn life for about three years. He often thought about killing himself. Eventually, his family convinced him to take a job at an anthroposophical farm, where he looked after the animals but continued to live a reclusive life. Initial psychopathological evaluation revealed a patient with moderately severe depression and a distinct autistic phenotype — a pattern present not only at the time, but also traceable back to the first decade of life. However, it is debatable whether an autism spectrum disorder diagnosis is warranted, since the D-criterion according to the DSM-5 (psychological distress and dysfunction) cannot readily be considered met due to the autistic characteristics. The structural characteristics of the full autistic phenotype are evident, however.

But how should clinicians tackle the different ontological phenomena in the presentation of people with autism. In this context, the term ‘ontology’ refers to the psychobiological core and neurobiological embodiment of a mental phenomenon. This links the phenomenon to the question of whether it can be deliberately changed in a given situation.

As a tertiary referral center for the diagnosis and treatment of adult ASD, we have developed a psychotherapeutic treatment approach for adults with ASD (18) which is presently being validated in the world’s largest controlled randomized multicenter psychotherapy trial in adult ASD (19). Since 2011 we also started a specialized in-patient treatment program for patients with adult ASD and comorbid psychiatric conditions (FASTER-IP; 20). In particular in this in-patient context, we experienced that the problems and features for which patients with ASD came to our specialized ward were manifold. In the early years of our treatment protocol, most interventions focused on psychoeducation and the recognition and acceptance of one’s own autistic personality type. At the same time, we focused on helping patients to find a niche in life in which they could thrive, given their specific strengths and weaknesses. However, in recent years, we have also realized that there are behavioral and cognitive patterns that occur in a similar way in many patients and which do not belong to the core properties of the ASD structure. To further complicate matters, there are medical conditions such as depression, anxiety, dissociation, psychosis and substance dependence, which exacerbate the core autistic phenotype.

In an attempt to make sense of this plethora of different phenomena, we have recently developed the SPC model as a heuristic tool to help us better understand and clarify the nature of the phenomena we encounter in classical therapeutic settings. In our clinical practice, the SPC model has proven to be a valuable heuristic tool, helping caregivers to better understand patients, distinguish phenomena, and target actions. It has also helped affected individuals to better understand themselves.

The main ideas of the SPC model have been published in a German-language book on the ontological status of mental phenomena (21). In this paper, I will describe the model as a heuristic tool for understanding and treating people with autism, as well as people in general.

## Nosological considerations – the SPC model in treating autism

The ontological and nosological status of the phenomena that psychiatrists and psychotherapists encounter is diverse. Consider, for example, the case of an extremely tall, slim person with a height of 213 cm who presents to a psychiatric service with depression, social withdrawal and a negative body image, as well as low self-esteem and having been teased at school. Likewise, consider the case of an autistic person with the same characteristics, but an average body height. In clinical practice, practitioners commonly encounter a mixture of phenomena where clinical symptoms are intertwined with a person’s structural features, problems, problem behaviors, and medical conditions. Often, neither the affected person nor the practitioner is aware that these are different ontological and nosological phenomena which should be dealt with differently depending on the nosological status. Ontologically, extreme body height and autistic personality are similar to phenomena such as hyperopia or visual acuity, which are unchangeable facts of life. Nosologically, they can represent variants of the norm if caused by multigenic factors or disease entities if caused by specific genetic or acquired pathological factors (body height: Marfan syndrome and acromegaly; autism: 22q11 syndrome and rubella encephalopathy). Problems such as bullying, debt, and problematic behaviors such as social withdrawal are not essential features of a given structure or disease, even though they tend to be linked to specific structures. For instance, people with autism often report being ostracized and bullied, and social withdrawal is a common response to unsuccessful interpersonal communication. Medical conditions such as depressive episodes, states of tension, and dissociation complicate matters and are often, though not exclusively, seen in people with autism.

The art of diagnosis involves analytically separating these different phenomena. The SPC model as a heuristic instrument may be helpful here (see **Figure 1**; **Table 1**). The acronym SPC stands for structure (S), problems or problem behaviors (P), and medical conditions (C).

**Figure 1:**
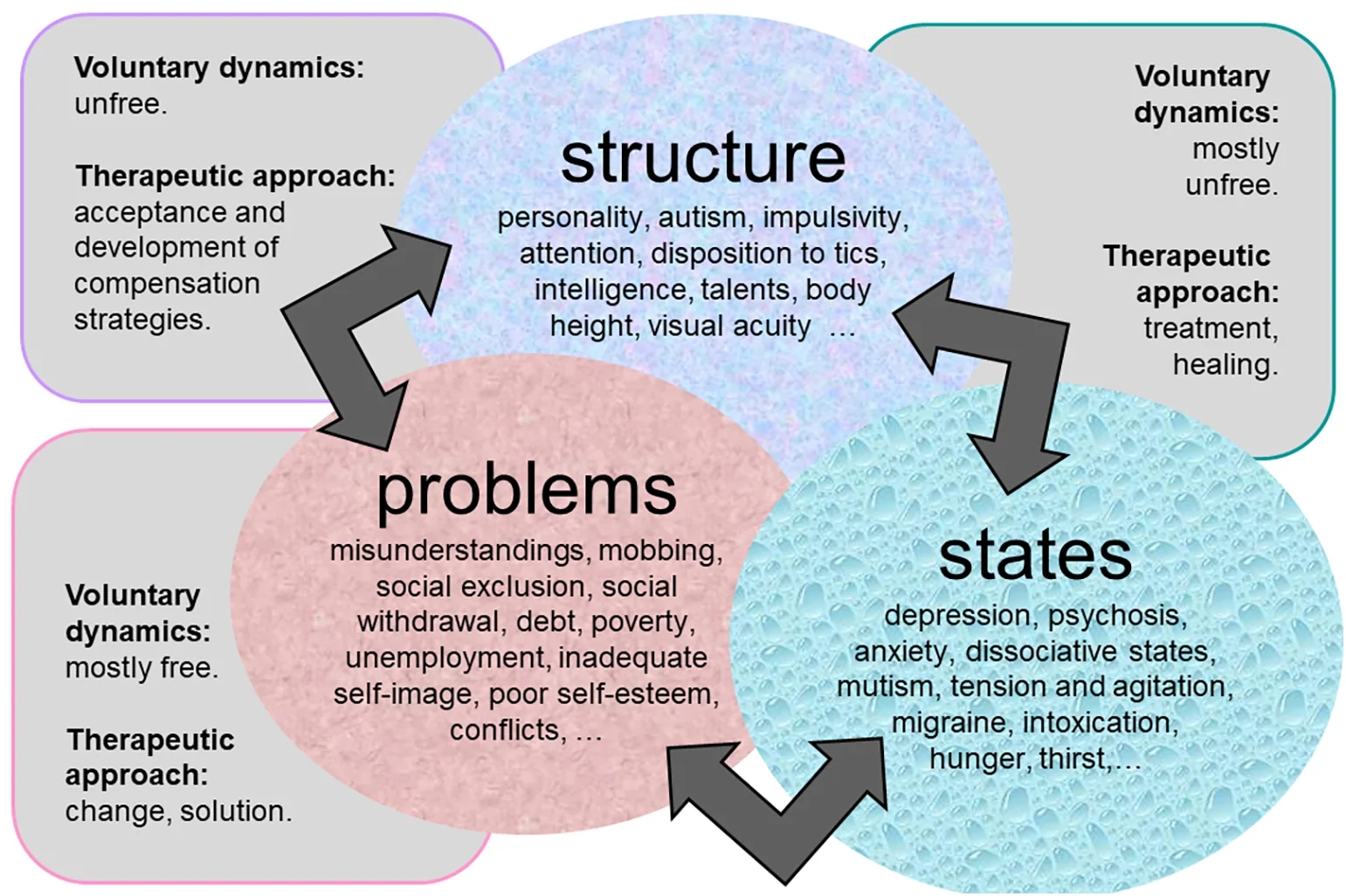
Illustration of the SPC heuristic scheme. Mental and somatic phenomena exist in different ontological categories. Structural phenomena, such as height, personality structure and autism, are chronic, essentially stable characteristics of the body. Psychosocial problems (e.g. misunderstandings, misperceptions and bullying) and problematic behaviors (e.g. social withdrawal, dysfunctional communication patterns and extreme identities) often develop in a typical way from structural specifics. Mental or physical conditions, such as stress, depression and dissociation, often emerge in specific problem constellations and can modify structural phenomena and behavioral patterns. The SPC model can help clarify whether a given phenomenon should be understood as a fateful structure, a solvable problem, or a condition that can be treated medically. Adapted and modified from Tebartz van Elst (2023) (3). All rights reserved W. Kohlhammer GmbH.

**Table 1:** Summary of characteristics, ontological status and therapeutic approaches towards structural phenomena in a therapeutic constellation.

Characteristics	Endurable phenomena; cannot be changed in a situation; are fateful properties of a body
Nosological status	May be norm variants but also sequelae of diseases (then handicap/disability)
Common constellations in psychiatry	Autism, personality structure, IQ, disposition to develop depression, psychotic episodes etc.
Common constellations in general medicine	Body height, visual acuity, hyperopia, myopia, disposition to migraine, seizures, hypertension etc.
Ontology: naturalistic organization, self-concept and changeability of phenomenon in question	Essentially unchangeable phenomena. Recognizing the ontological status of the phenomenon as destiny, perhaps task, challenge. Phenomenon is not linked to one’s own responsibility, guilt, wrongdoing, etc. Individual is not free in relation to the phenomenon in question.
Therapeutic and self-approach	Acceptance of traits as a fateful phenomenon by patients, relatives, friends, partners and caregivers. Development of compensation strategies. Construction and identification of ecological niches.

Structural phenomena are characterized by the fact that they are inert by nature and cannot be influenced by free will or effortful control in a given situation. A classic example of a structural phenomenon is body size. This must be understood as an inherent property of the body, for which the affected person is not responsible. Such phenomena should be regarded as fate, and the affected person must learn to cope with them. Most aspects of autism must be understood in terms of structural properties. For example, a person’s compromised theory-of-mind (TOM) competence is not under their control, but must be understood as a strength or weakness similar to visual acuity. Autistic people cannot arbitrarily modulate their aversion to looking other people in the eye. The same applies to the high sensitivity to socially complex stimuli experienced by many autistic people. One reason why many people with autism perceive the diagnosis as a relief is precisely because of this. This is because, when someone is diagnosed with autism, these phenomena are recognized as inherent and unavoidable personality traits. Previously, they were considered misbehavior by the affected individuals or their significant others, and the affected individuals or their environment were held responsible.

When dealing with patients, it is important to recognize these structural peculiarities. In everyday language, these are usually referred to as strengths or weaknesses. These include easily identifiable physical characteristics, such as height or visual acuity. However, mental characteristics such as general intelligence, perceptive peculiarities such as sensitivity to noise and overload, and complex mental characteristics such as the aforementioned Theory-of-Mind capacity must also be understood as structural, innate characteristics of a person. This is because they cannot be changed at will by the affected individuals. **Table 1** summarizes the definition of this concept of personality structure and provides examples.

In the SPC model, the ‘P’ stands for ‘problems’ and ‘problem behaviors’. Problems frequently observed in people with high-functioning autism include bullying and exclusion. These often result from misunderstandings by others. For example, a tendency towards direct communication combined with a reluctance to socialize in groups may be misinterpreted as arrogance. Social exclusion is a common reaction to such a misperception. Another typical problem behavior in autistic people is social withdrawal. Many people with autism avoid social situations because they repeatedly struggle with group communication. Consequently, they prefer to withdraw into their own world and avoid social contact. However, social exclusion, bullying and social withdrawal cannot be considered structural phenomena. Rather, they are problems or problematic behaviors. Unlike structural phenomena, which should be accepted as part of one’s body composition, problems must be solved and problematic behaviors changed (see **Figures 1**, **2**; **Tables 1**–**4**).

**Figure 2:**
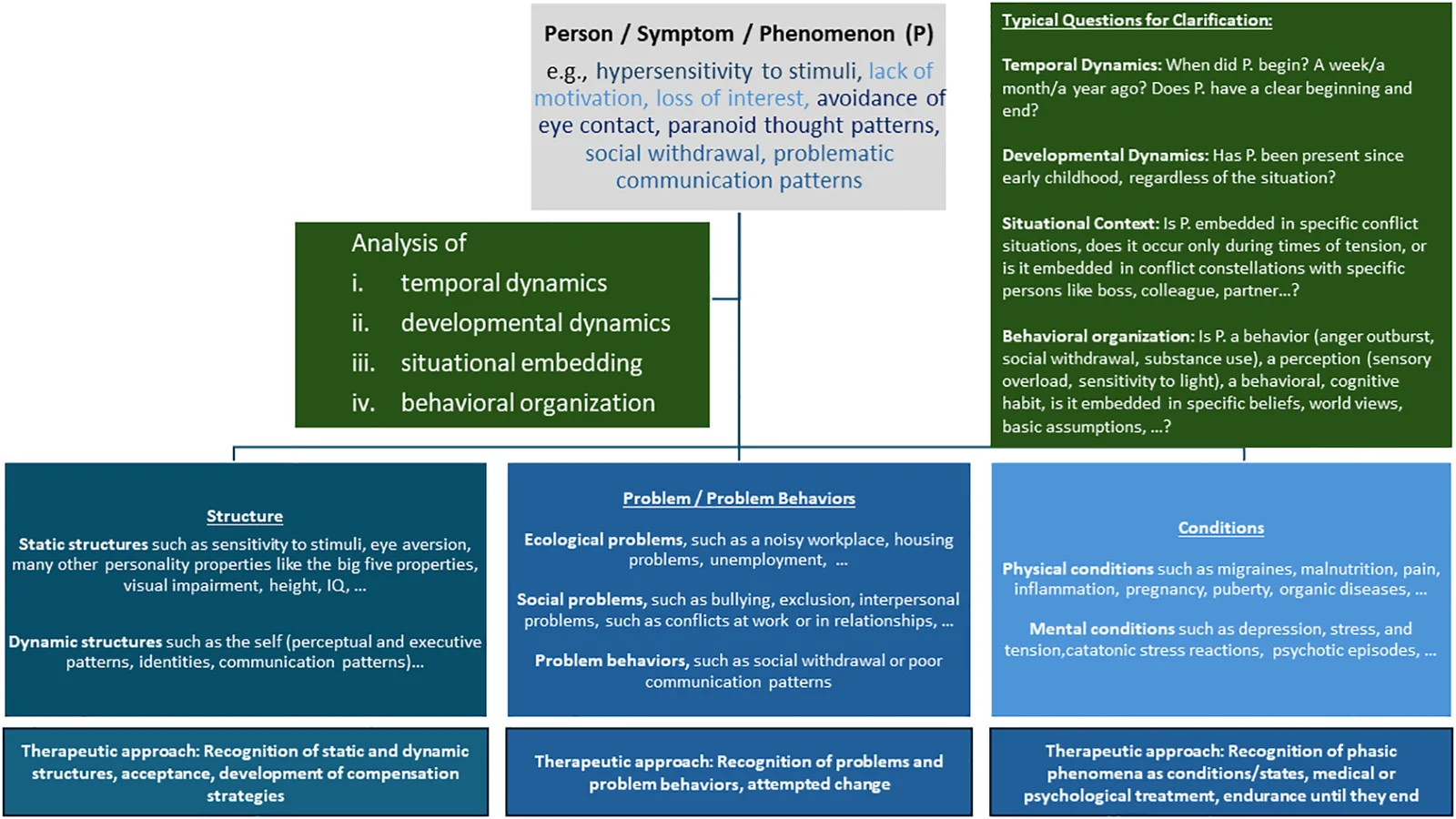
This flowchart illustrates a potential approach to determining the ontological status of the various phenomena encountered by therapists in clinical practice. Based on its temporal or developmental dynamics, situational embedding, and behavioral organization, a given phenomenon can be assessed as a structural (unchangeable), situational (unchangeable but transient), or problematic (solvable) entity.

**Table 2:** Summary of characteristics, ontological status and therapeutic approaches towards problems and problem behaviors in a therapeutic constellation.

Characteristics	Variable, situational phenomena; imposed by the environment or others; acquired dysfunctional behavioral patterns
Nosological status	Problems and problem behaviors are not diseases
Common constellations in psychiatry	Mobbing, social exclusion, deprivation, unemployment, poor housing, poverty, social withdrawal, drug abuse, aggressive behavior, dysfunctional communication etc.
Common constellations in general medicine	Poverty, poor hygiene, poor housing, exposition to environmental toxins; eating habits, drinking habits, hygienic habits, communication habits, smoking, etc.
Ontology: naturalistic organization, self-concept and changeability of phenomenon in question	Changeable phenomena. Individual has potential to work on change, to change behaviors, communication patterns, etc. Phenomenon belongs to the realm of personal responsibility and free will. Misbehavior is possible.
Therapeutic and self-approach	Phenomenon should not be accepted per se. Psychosocial problems should be solved if possible. Dysfunctional behaviors should be analyzed, alternative behaviors should be considered and trained.

**Table 3:** Summary of characteristics, ontological status and therapeutic approaches towards mental and physical conditions or states in a therapeutic constellation.

Characteristics	Phasic or episodic phenomena do have a beginning and an end represent functional states of the body which can change not in the situation but in time
Nosological status	May be physiological reactions, norm variants but can also be expression of a disease
Common constellations in psychiatry	Depressive episodes, episodes of stress and anxiety, sensory overload, psychotic decompensations, states of intoxication, anorexia, inflammatory brain states etc.
Common constellations in general medicine	Migraine attack, epileptic seizure, inflammatory states in autoimmune diseases, pregnancy, starvation, etc.
Ontology: naturalistic organization, self-concept and changeability of phenomenon in question	Potentially changeable phenomena. Body can switch back to original, more desirable state of functioning. State change cannot be induced voluntarily, but is indirectly supported by medication, psychotherapy, behavioral changes.
Therapeutic and self-approach	Possible medical or psychological treatments should be identified and tried. The therapeutic goal is to restore the desired and better medical condition or state.

**Table 4:** Summary of different concepts of the term structure in philosophy, psychoanalysis, developmental psychology, sociology, and biology (modified according to 21–26).

Different scientific concepts of structure	Definition and theme of the concept of structure	Conceptual similarities and specificities
General Definition of the Concept of Structure	The term ‘structure’ refers to the properties of complex living or non-living systems, their sub-components and the relational connections between these sub-components. These properties are inert and, if they can be modulated at all, it is only slowly.	All structural concepts have the following in common: a larger whole; subcomponents; organized relationships between components; the whole being more than the sum of its parts.
Philosophical concept	Depending on the subdiscipline, topics in philosophical concepts of structure include being (ontology), knowledge (epistemology), thought (logic), science (philosophy of science), language (philosophy of language), etc.	Structural concepts are typically highly abstract and, in some instances, challenging to verify empirically.
Psychoanalytic concept	The term ‘structure’ refers to the organization of the personality based on relational experiences during critical formative phases, such as the oral, anal, phallic, Oedipal, latency and genital phases, as well as the organization of the psychological apparatus (e.g. the ego, the id and the superego). More recent formulations focus more on structural capacities such as self-development, affect regulation, identity formation and relationship-building.	Empirically assessable developmental dynamics are examined on an individual basis, focusing on parent-child relationships, against the backdrop of robust theoretical frameworks.
Concept in developmental psychology	Developmental psychology examines the structures of perceptual, motor, emotional, linguistic, cognitive, and social abilities, the dynamics of their development, and how these individual components are interrelated.	The focus is on empirically measurable mental abilities (perception, eye contact, motor skills, language development, theory of mind, etc.). The structure has a certain developmental dynamic.
Sociological concept	Sociology examines the structural patterns of groups, societies and states, as well as their characteristic components and regular relationships within the organization of society as a whole.	The focus is on large-group dynamics rather than on the individual or interpersonal structure of mental processes.
Biological concept	In biology, the structural characteristics of molecules, cells, tissues and organisms are described. These structures are usually associated with identifiable functions.	For example, the structure of bone ensures mechanical stability, while the structure of muscles enables movement.
Static biological structures	Static biological structures change very little outside of the developmental phase, remaining stable throughout the lifespan except in the context of specific disease processes.	Classical somatic example: body height, hyperopia. Classical mental example: IQ, sensory hypersensitivity and problems with theory of mind in autism
Dynamic biological structures	Dynamic biological structures are situationally stable but can adapt to changing requirements through gradual adjustments.	A classic somatic example is muscle mass. A classic mental example is the ability to play instruments, read and write, and suppress impulses. Other examples include worldviews, core beliefs, the self and identities.

Finally, conditions represent the body’s true functional modes, which are rooted in its physiology and sometimes pathophysiology. Due to the physiological cybernetic organization of the complex human body, and in order to enable it to adapt to different environmental and situational circumstances, evolution has created various functional modes of the human body. Examples include wakefulness and sleep, nutritional and digestive conditions (such as hunger, thirst and satiety), the resting state, the fight-or-flight state, sexual activity, puberty and pregnancy. All of these physiological functional states of the human body may be overactive or underactive. The underlying cybernetic neurophysiology may be due to normal genetic variance in individual functioning. In this case, it should be considered a variant of the norm. However, it may also be caused by genetic or acquired disease processes. In these cases, the respective over- or underactivities should be considered as disease processes from a nosological perspective. These processes are often modifiable by medication, as in the cases of inflammation, headaches, depression and psychosis. The appropriate ‘medication’ for hunger and thirst, of course, is eating and drinking. Thus, the phenomenology and physiology of alternating medical conditions should be regarded as fundamentally normal and adaptive organizations of the human body as they have developed through evolution.

In this sense, classical mental conditions are states of heightened awareness and concentration in fight situations, relaxed drowsiness in recreational situations, narrowed awareness in reproductive situations, focused aggression when offspring are threatened. Depression, tension (stress), dissociation, anxiety (panic attacks) and paranoid episodes are further examples of such mental conditions. Conditions differ from structures in that they are functional, evolutionary and adaptive by nature, with a definable beginning and end. In contrast, structures are chronic and unchangeable.

Classical bodily conditions include migraines, systemic inflammation (e.g. when suffering from flu), anorexia or starvation, and physiological states such as pregnancy.

It is important to recognize that such conditions may modify a person’s structural peculiarities as well as the dynamics of problematic situations.

For example, autistic people in chronically stressed or depressed states commonly exhibit a phasic deterioration of the autistic phenotype rather than recognizable classical symptoms of depression, such as depressed mood, loss of interest, anhedonia, and loss of drive. Instead, when depressed, they become more sensitive to situations that overwhelm them, more rigid, and more dependent on daily routines. They are less capable of compensating for poor eye contact, unmodulated prosody, and reduced gestures and facial expressions. For professionals unfamiliar with the dynamics summarized and illustrated in the SPC model, such phenotypes may simply appear to be more severe autism, as a structural property of that person. However, this interpretation overlooks the fact that, as a structural diagnosis, autism is a chronic and fundamentally stable condition, and that the diagnosis of autism itself cannot explain why, in this individual, there is a phasic deterioration of the classical autistic symptoms when, earlier, the person was perfectly capable of coping with their stable autistic characteristics. This exacerbation of autistic symptoms is better explained by co-occurring depression, which is often stress-induced.

Diagnosticians must be aware that a diagnosis of depression may present atypically in people with autism, appearing as an exacerbation of autistic symptoms. However, a depressive episode is neither a chronic personality trait nor a situational problem or behavior. Rather, it is a physical state linked to the pathophysiology underlying such states. This knowledge is crucial for therapy because it implies that this additional severity of autistic symptoms should not be regarded as a structural phenomenon that must be accepted and compensated for. On the contrary, depressive states should be treated with all available means, including psychotherapy and pharmacotherapy.

The same is true of other states, such as starvation, anorexia, anxiety, panic and psychotic decompensation, as well as migraine, chronic inflammatory states and pain, and physiological states such as pregnancy. Unlike structural phenomena, state-like phenomena have a beginning and an end. They should not be regarded as a natural aspect of one’s personality. They cannot be changed like a problem can be solved. Instead, they should be treated and healed whenever possible. This applies to the state experienced during a migraine attack, as well as to a depressive episode or a psychotic decompensation experienced by a stressed autistic individual. **Table 3** summarizes the different nosological characteristics of state-like diagnoses and provides examples.

## Unravelling the psychodynamics of everyday life: the therapeutic benefits of the SPC model

In autistic individuals, a typical psychodynamic often emerges within the three poles of the SPC model. For example, limited Theory of Mind (TOM) skills, combined with a direct communication style that lacks pragmatism, and restrained facial expressions and gestures, can lead to them being perceived as arrogant, overbearing, egocentric, selfish and pitiless, particularly among highly functioning patients. This misperception can lead to negative countertransference and reactions from third parties, such as social exclusion and bullying. While reduced TOM ability must be understood as a fateful structural phenomenon, this is not the case for problematic phenomena such as bullying and social exclusion. The problematic behavior of social withdrawal that often develops in such situations should not be considered an integral part of the structural characteristics of an autistic syndrome. Rather, it is a behavior that can usually be understood, but which should be treated therapeutically and ideally changed.

When high-functioning autistic people try desperately to camouflage themselves and adapt to their environment but do not succeed despite all their efforts, chronic overload often leads to depressive or even psychotic decompensations. However, as mentioned above, depression can sometimes resemble an exacerbation of autism. The typical autistic stress reactions and decompensations, such as shutdowns (mutism, freezing and dissociative episodes), may lead to a misdiagnosis of borderline personality disorder. These reactions should also be evaluated as state-like decompensations, which are typically triggered by situational stressors and problematic situations. Too much sensory input, small talk, or disruption to expected routines, for example, may trigger dissociative states of mutism and stupor. Subsyndromal variants of such stress reactions resemble more autistic behavior, even though these phenomena are state correlates and not structural features. **Figure 1** illustrates this relationship.

The therapeutic benefit of the SPC model is that it helps both patients and therapists develop a more accurate self-perception and understanding of symptoms, and to distinguish intertwined everyday phenomena analytically.

This is important because it has far-reaching and negative therapeutic consequences when structural, problematic, and medical conditions or states are not recognized properly. For example, if a structural phenomenon such as a person’s compromised TOM capacity or sensitivity to sensory overload is mistaken for a problem, the implicit therapy goal will be to solve this pseudo-problem. However, given that this is a structural property of the patient’s body, it is impossible to change this deficit. By choosing to resolve poor TOM faculties or sensory overload sensitivity as a therapy goal, the implicit message to the patient is: ‘The way you are is not OK and should be changed.’ This undermines the patient’s self-image and self-esteem. At the same time, such a therapy goal is unachievable and will inevitably lead to frustration and despair. The aim of psychotherapy should not be to dissolve the structural properties of a person’s body. It is extremely important in the psychotherapeutic process to identify and practice compensation strategies for core structural weaknesses. However, they often require the person concerned to recognize and accept their structural characteristics. This is central to establishing a valid self-model and stable self-esteem. As wearing glasses is a successful compensation strategy for many people with ametropia, acquired and learned communication strategies can be highly effective in preventing misunderstandings in individuals with autism and limited Theory of Mind (ToM) abilities. Just as glasses do not heal myopia or hypermetropia, successful compensation strategies do not eliminate autistic properties.

Problems and problematic behaviors, sometimes referred to as challenging behaviors, should not be confused with fateful structures. Doing so would lead to the erroneous assumption that they must be accepted as inevitable phenomena. Social withdrawal, for instance, is a common response to repeated failure in interpersonal relationships. Social withdrawal is often an understandable avoidance behavior in the face of the fear of repeated failure. Nevertheless, it remains a problematic behavior because it leads to social isolation. The same applies to dysfunctional communication patterns, which are often observed in people with high-functioning autism. If such phenomena were misinterpreted as inevitable, this would lead to loneliness and social isolation. Misinterpreting problem behaviors as inherent characteristics that must be accepted limits the potential for the development of autistic people, as it means that existing development prospects are deemed impossible.

Finally, if the phasic deterioration of an autistic phenotype is misinterpreted as a structural phenomenon rather than recognized as an expression of a stress reaction or possible subsyndromal depression, the option of antidepressant treatment is overlooked. This is because stress reactions and depression can manifest in autistic people as an increase in the severity of autism, rather than classic melancholic depressive symptoms. However, this should be addressed using traditional psychiatric and psychotherapeutic methods, rather than being accepted as inevitable.

This can also be illustrated by the classic autistic stress response. The term ‘overload’ usually describes a primarily structural phenomenon. For example, too many stimuli, excessive noise, sensory contact, too much metaphorical language, social situations or frustrated expectations can be significant triggers for stress reactions, and this can be unexpected for some people. This refers to a fateful, structurally determined vulnerability.

The term ‘shutdown’ describes a phasic stress reaction with a beginning and an end. This can lead to dissociative reactions, such as changes in perception, loss of bodily sensation, reduced pain perception, and impaired associative ability, or catatonic reactions, such as mutism, freezing, thought emptiness, motor stereotypes, and aggressive behavior. However, as with migraine attacks, these phenomena are temporary, have a natural end—unlike structural phenomena—and can be treated with medication, skills, or psychotherapeutic methods. Finally, it is important to distinguish between problems and problem behaviors, and structural or situational phenomena. This is because the scope of behavior is greatest in this phenomenological area. Problems and problem behaviors can be solved by learning how to deal with them. Misattributing them can lead to a narrowing of the behavioral and developmental scope of autistic children, adolescents and adults, thus obstructing positive development prospects.

In clinical practice, it may be difficult to determine at first glance whether a given symptom should be considered a structural, problematic, or a condition-like phenomenon. **Figure 2** illustrates a potential flowchart showing how clinicians might address this problem and clarify whether the phenomenon of interest should be accepted and compensated for, solved and changed, or treated/endured like similar medical conditions experienced by non-autistic people.

## Discussion

The SPC model is a heuristic model. It can help those affected and therapists to better understand the natural constitution, freedom and modifiability of mental phenomena. In positive cases, this can lead to greater acceptance of structural phenomena, motivation to change problematic behaviors, and serenity and confidence when facing adverse conditions.

What is new about the SPC model, and how does it differ from existing models in developmental psychology and psychiatry? This question will be discussed briefly here in comparison to the classic biopsychological model of psychiatry: the diathesis-stress model of mental disorders including autism (27–29), which distinguishes between predisposing genetic and other unchangeable factors (diathesis) and situational and contingent factors (stress) in the causation of mental disorders (conditions). While certain parallels can be seen here — in that the structural pole is closely related to diathesis, the problem area to stress and the conditions to mental illnesses such as psychotic or depressive episodes — the SPC model is more granular. It addresses the ontological nature of the various poles in a more nuanced way. For example, structure is not initially understood as a pathological concept. A clearer distinction is also made between phasic phenomena (states) and chronic phenomena (structures). Both may represent as physiological or pathological phenomena. Furthermore, the pattern-like nature of psychodynamic relationships between structures (e.g. body-height, IQ, ASD or ADHD personality structure) and typical problem patterns (e.g. bullying, social withdrawal, dysfunctional communication, problems with the self) is examined with greater analytical clarity. In light of the growing criticism surrounding the overdiagnosis of developmental disorders such as ADHD and ASD, one of this model’s strengths is its ability to effectively capture the psychodynamics of subsyndromal structural diagnoses (i.e. broader autism/ADHD phenotypes). This is because psychological distress and dysfunction often stem not from the structure itself, which can be more or less pronounced, but from the problems that develop as a result, such as social exclusion, and the problematic behaviors that emerge, such as social withdrawal, inaccurate self-images and overcompensated identities. Compared to other developmental psychological models, such as those of Erikson, Havighurst or Piaget, the model’s lack of theoretical complexity is a further advantage, as it allows for greater flexibility and generalizability.

The SPC model could also help to resolve the growing controversy surrounding the neurodiversity discourse. In this context, one might refer to the notable statement by Uta Frith, a leading figure in autism research. In a recent statement, she declared the concept of the autism spectrum a failure due to its ever-widening scope (30). As the SPC model’s concept of structure is not inherently pathological, but rather aims to account for physiological variance in biopsychological phenomena such as impulsivity, inattention, IQ and autistic traits, the model can now also qualitatively account for sub-syndrome structural phenomena, such as a broader autism/ADHD phenotype. This is because typical interpersonal and social dynamics often differ only slightly above and below an imaginary threshold for a dimensionally structured personality.

### Further refinement of the structural concept

Within the context of clinical communication and the theoretical discourse on the concept of structure as presented in the SPC model, it quickly becomes clear that, although the concept of structure is intuitively accessible and easily understood by patients in everyday language, it must also be further differentiated theoretically. The concept of structure is used as a key concept in psychoanalysis, developmental psychology, biology, sociology and philosophy, but these disciplines often refer to different phenomena and details when doing so. The most common scientific operationalizations and contents of structure concepts are summarized in **Table 4**.

### Limitations and constraints of the model

It must be stressed that the SPC model is presented as a heuristic tool derived from clinical practice. It has not yet been empirically validated, so future research should assess its impact on patient outcomes, the therapeutic alliance and clinical decision-making processes.

Also, in the case of autism, even experienced experts cannot always immediately determine whether certain phenomena, such as the biographical self-concept, are structural or problematic. The same applies to identities that appear structural from the perspective of a single situation because they cannot be changed in that situation, yet can be changed over longer periods of development. It is therefore sensible to further distinguish between static structures, such as body size, visual impairment, IQ or sensory hypersensitivity in autism, and dynamic structures, such as the self, identity, worldviews and core beliefs (see **Table 4**). For when analyzing the clinical reality of many autistic people it becomes clear that different aspects of structural mental phenomena are intertwingled. For example, a person with autism may experience stress at a cocktail party due to auditory overload, the multitude of social demands and the difficulty of understanding sarcasm, jokes and non-literal communication. This could lead to the development of an inferiority complex or feelings of systematic discrimination and intentional exclusion by others, even if these were not objectively present. Any subsequent aggressive outbursts would be viewed as problematic behavior within the SPC model. Sensitivity to sensory overload and communication difficulties would be viewed as fixed, static structural characteristics. Conversely, the concept of feeling disadvantaged or having an inferiority complex should be understood as a dynamic phenomenon, even though it is situationally unfree. It is important to differentiate between static and dynamic structures because, even though dynamic structures cannot be changed from the perspective of a single situation, they can still be addressed in therapeutic processes and may change over time, albeit very slowly. Therefore, dynamic structures occupy an intermediate position between the ‘structure’ and ‘problem’ poles of the SPC model (**Table 4**).

Finally, the SPC model is still just a model. It is a heuristic tool that does not describe reality itself. Similarly to the three-instance model of psychoanalysis, the human body does not contain three different substances representing the ego, the id and the superego, but rather one body that encompasses everything. However, clinical experience shows that the model can be helpful in promoting self-awareness and in distinguishing the different ontological constitutions of mental phenomena that initially appear to be similar. Furthermore, it can facilitate the therapeutic process by enabling a joint examination to establish whether the intended therapeutic goal is structural, problematic, or due to specific conditions.

Furthermore, discussions initiated by Uta Frith regarding the expansion of the ASD concept demonstrate the need for a conceptual framework for the dimensional, structural organization of the autistic phenotype from clinical, therapeutic and scientific perspectives. This is because neurobiological studies also suggest that autism is not a condition that is either present or absent, but rather a set of neurobiological information-processing patterns that can vary in intensity and are therefore dimensional in nature (31, 32).

Finally, quite independently of autism, the SPC-model can also be helpful in understanding other developmental disorders (e.g. ADHD, intellectual disability, and tics), as well as the everyday behavior of other people, one’s own caregivers, and one’s own body.

Future studies should empirically demonstrate the clinical and conceptual benefits of the SPC model. To this end, a qualitative research approach could initially be employed to survey therapists and patients on the subjective advantages of this concept. Further, more systematic studies could investigate whether the model helps those affected to develop more appropriate self-concepts and identities.

In summary, this paper presents a heuristic model developed in recent years in the context of autism therapy, in which mental phenomena are differentiated according to their psychobiological ontology. It distinguishes between unfree structural phenomena, which must be accepted as fateful chronic phenomena and psychobiological conditions, which are temporary in nature. Finally, problems and problem behaviors that develop in a patterned way are identified in the psychodynamics of everyday life. This area offers the greatest potential for action and the greatest room for individual development in terms of creatively and constructively shaping one’s own life.

## Data Availability

The original contributions presented in the study are included in the article/supplementary material. Further inquiries can be directed to the corresponding author.
